# Reality or a Dream: Barriers and Facilitators for Nursing Assistants Pursuing a Nursing Career

**DOI:** 10.1093/geroni/igab046.3730

**Published:** 2021-12-17

**Authors:** Stephanie Trotter, Leah Richardson

**Affiliations:** University of Arkansas for Medical Sciences, Little Rock, Arkansas, United States

## Abstract

Certified nursing assistants (CNAs) serve a critical role in the care of older adults. However, CNAs often experience significant professional and personal burdens related to caregiving work. Professionally, CNAs experience exorbitant workplace stress (e.g., physical injury, burnout, emotional exhaustion, staffing shortages, turnover). Personally, CNAs may have only a high school education, are subjected to low-paying jobs, and little opportunity for career advancement. Further, CNAs are disproportionately of minority race. Clearly, CNAs are negatively impacted by many social determinants of health. Ultimately, these burdens negatively impact older adults’ care provision and quality of life. Transitioning to a nursing career may alleviate some of these complex problems, but this has scantly been explored. A qualitative descriptive study was designed to 1) identify interest in a nursing career, and 2) explore perceived barriers and facilitators of transitioning into a nursing profession. CNAs from nursing facilities participated in private, semi-structured interviews. Recorded interviews (n = 6) were transcribed verbatim. Preliminary thematic analyses yielded two overarching themes: The Dream and The Reality. Rich subthemes began emerging from both overarching themes. Example subthemes from The Dream were: family legacy in healthcare, and finding purpose. Example subthemes from The Reality were: versus (CNAs vs. nurses; nurses vs. nursing care), and work-life balance. These preliminary findings suggest that CNAs express desire in becoming a nurse, although a range of personal and professional barriers and facilitators exist. Making the nursing dream become reality may improve CNAs’ social determinants, workplace outcomes, and resident outcomes, but further exploration is warranted.

